# Coworking and Coliving: The Attraction for Digital Nomad Tourists

**DOI:** 10.1007/978-3-030-65785-7_17

**Published:** 2020-11-28

**Authors:** Ekaterina Chevtaeva

**Affiliations:** 1 Department for Informatics, Technical University of Munich, Garching bei München, Bayern Germany; 2 Smart Tourism Education Platform (STEP) College of Hotel and Tourism Management, Kyung Hee University, Seoul, Korea (Republic of); 3 Department of Tourism and Service Management, MODUL University Vienna, Vienna, Wien Austria; The School of Hotel and Tourism Management, Hong Kong Polytechnic University, Kowloon, Hong Kong SAR, China

**Keywords:** Digital nomad travel, Coworking and coliving space, Travel experience, Coworkation

## Abstract

The study facilitates digital nomadism for tourism research and recognizes a unique product offer on the market: the combined coworking and coliving space in compelling or exotic destinations. The aim of the study is to explore the experience of coworking and coliving by digital nomads and identify valuable elements. Qualitative interview data are used to analyse combined coworking and coliving space environments from the perspective of digital nomad tourists. A better understanding of digital nomad preferences may help destinations and business owners to attract digital nomads during and after the pandemic. The study’s findings, perceived advantages and disadvantages of coworking and coliving spaces, may serve as a guideline for targeting digital nomads.

## Introduction

Numerous coworking hotspots worldwide attract digital nomads; some examples include Bali, Lisbon, Mexico City, Barcelona. A more recent trend is in work-travel coliving spaces as a part of the coworking in exotic environments or places promising exploration of a new place or benefits of warm weather, beaches, or a calm environment for recreation [1]. This trend may also be known as “coworkation,” an inspirational coworking retreat set in stunning locations around the world [2], within a coworking and coliving space. Some countries see the potential in digital nomad tourism to restart the economy after the pandemic crisis of 2020 by introducing an attractive visa policy for remote workers [3].

The destinations and hospitality industry may benefit from gaining a better understanding of the role of coworking and coliving for digital nomad tourism. It is worth opening a conversation about the new type of attractions to facilitate digital nomad travel’s institutionalization for tourism research. This study aims to explore the experience of a combined coworking and coliving space during travel and acknowledge elements that digital nomads most value. The study questions which factors affect digital nomad’s choice to stay in the coworking and coliving space.

## Overview of Coworking and Coliving

Coworking and coliving space belong to the sharing economy, where the value is co-created [4, 5]. The majority of the previous studies recognized the value of bonding with like-minded individuals as the main advantage of coworking and coliving space [6–10]. Digital nomads tend to group within like-minded communities of location-independent individuals [11]. Therefore, collective work-leisure hubs that combine working and living facilities are likely to foster guests’ interpersonal relationships [8]. Nevertheless, only a few studies distinguished travelers from local users of coworking spaces or focused on coworking as a part of the hospitality industry.

Establishing of coliving spaces was a market response to the labor conditions crisis that later influenced the conventional meaning of home, making it a co-productive and co-emergent practice: instead of being an escape from work, it is productive; instead of being private, it is social; and instead of being inhabited long term, it is mobile [5]. The proposition of coliving space is in providing a ready-made community to its residents, providing a sense of belonging in the disconnected location [12]. Creating this sense of community is essential for digital nomads’ wellbeing [9]. Besides community and flexibility, one of the main features that make coliving attractive is that the culture of sharing leads to a reduction in the cost of living and travelling [13].

Nevertheless, for digital nomads in travel destinations, coliving space as part of a coworking space commonly has a one-week minimum stay requirement, making it a less flexible option than room renting or a hotel. The majority of the coliving spaces in inspiring destinations target digital nomads in their marketing activities, and it reflects the new meaning of a home through multiple short-term tenancies across the globe, home-as-work, and home as a social network [5]. One of Bali’s coworking spaces with coliving accommodation indicated that 90% of its customers refer to themselves as digital nomads [14].

## Methodology

This study investigates the experience of staying in coworking and coliving space during travels following a qualitative research design by using inductive content analysis. Semistructured interviews were completed online in May 2020 to collect data for the study. Participants were asked to reflect on their experience in coworking and coliving space, share what they would have improved and future travel preferences. The participants’ search was performed via social media accounts of different coworking and coliving companies and targeted travelers who experienced them abroad. The principle of saturation was applied to distinguish the sample size. In total, 12 participants were involved in the study, representing five nationalities, covering coworking and coliving experience in Asia, Europe, Africa, South, Central and North America. Data collection and data analysis were conducted simultaneously. The data was analyzed by one researcher, which is common for inductive content analysis [15].

## Findings and Discussion

This section summarizes ten main themes drawn in regards to coworking and coliving experience. The most recognized advantage is a community sense that includes easy access to meeting people and events; this finding is aligned with previous research [6–10]. In contrast to the community spirit, three participants indicated that they felt the lack of privacy at the same time:

*“There is sort of that social pressure, people want to talk to you. There is this sort of expectation that people are friendly and want to network. [. . .] I guess I would make it a little bit more private.”*

The main perceived advantages and disadvantages of coworking and coliving spaces were noted and summarized in Table 1. The next thread is convenience that combines such elements as the location of coworking and coliving spaces close to each other, location near restaurants or the beach, food availability, extra services (pick up from the airport, pre-arranged sim card, assistance with renting transportation), and workspace availability 24/7. This theme is aligned with previous studies, as several researchers recognized the level of accessibility valued by digital nomads [6, 16]. Moreover, Bendkowski [17] separately distinguished food and beverage service, 24/7 building access, and other dedicated services. However, it is essential to point out that not all coworking spaces are the same, and some participants shared experience about coworking places that were closed on weekends (mostly in Europe). Some coworking places only had a furniture and internet connection with no other services. Also, five participants shared that it is challenging to find a coliving space in most locations.

**Table 1.** Advantages and disadvantages of coworking and coliving spaces during travel.

Advantages	Frequency	Disadvantages	Frequency
Community sense	11	Limited availability of coliving (limited destinations)	5
Pleasant work environment	7	Party scene	5
Business advice & place to learn from others	7	Expensive compared to a similar quality of accommodation	4
Convenience	5	Segregation from the local community	4
Helpful with local tips	4	Lack of privacy	3

Interestingly, the advantage of convenience comes with a price; segregation from local people and culture. Four participants mentioned distancing from a local community, with one person comparing coworking and coliving space with a Disneyland experience. Digital nomads being separated from locals was also found in previous studies [6, 18].

Another recognized area is the pleasant work environment of the coworking space. Participants pointed out such features as comfortable furniture, good lighting, plants, air-conditioning, light, noise level, internet speed, and coffee quality. The importance of the atmosphere and interior aesthetics and the layout of the space was previously mentioned by researchers [19]. Besides a pleasant work environment being acknowledged as an advantage, five participants mentioned a negative experience in coworking and coliving places with loud parties being a major distraction from work. These findings suggest that calling a location “coworking and coliving space” potentially has different meanings to different people. The definition of “coworking and coliving space” may vary: between a simple place with Wi-Fi and a full-service hotel; between a quiet place to collaborate on work and a place of entertainment. This suggests a necessity for business owners to include elements that help to identify their purpose and gain trust – online promotions and recommendations, word of mouth, reviews, recognized brands and others.

The following theme emerged is that coworking space gives business advice and is a place to learn from others; this advantage was mentioned by Capdevila [20] when he highlighted that coworking spaces have a knowledge sharing dynamics that are often absent in shared offices. On top of sharing practical knowledge, participants learned about remote culture and slow living style.

An additional key issue recognized is related to cost; the price of the coliving option being higher than other accommodation types of the same quality. As previous literature revealed, for a coliving concept, community, flexibility and price are among attractions [13]. However, for travelers, flexibility is already limited by a minimum stay requirement, and by having a higher price than alternatives, the second common advantage of a coliving space is absent. It affects the competitiveness of coliving accommodation as part of coworking space on the market and contradicts the accepted perception of the shared economy being more affordable than private accommodation.

However, some participants saw a positive influence of a higher price:

*“Coliving I stayed in had a slightly higher price point, which I think helped filter out (people) [. . .] I think the higher price point sort of filtered out some of the people who were more on like “I don’t really know what I’m doing, sort of backpacker type of vibe.”*

Others perceived the facilities being overpriced. One shared:

*“I’ve only been in coliving space once but only to try it I didn’t like it because it was so expensive.”*

A different participant explained his/her reasons to moving to a hotel:

*“I was there for about a month and a half when they started doing coliving and I went to view the houses (coliving). It was more expensive to stay in a coliving space then to stay in a hotel. And that was ultimately the factor.”*

The next thread recognized is that coworking and coliving spaces provide helpful local advice, especially when first time in the destination. This was an exciting finding, as, besides a vast online community within remote workers who travel, many participants shared that they do not plan much in advance and refer to coworking and coliving spaces to find places to eat and things to do. From the tourism perspective, this may be a new opportunity for local businesses to recognize coworking and coliving spaces as information intermediates for travelers and establish partnerships within locals.

One more advantage is in the pulling ability of coworking spaces to attract digital nomads. Out of all participants, three mentioned that they are looking specifically for a combined coworking and coliving space in the destination, even choosing a destination based on it. It is an exciting new finding for destination managers and a new opportunity for emerging destinations. Surprisingly, these three participants were the most experienced travelers.

Another matter that emerged is in different perceptions of the digital nomad community by travelers from Asia. As most digital nomad travelers are Western, Asian nomads, when traveling, had a different community and travel experiences. Asian travelers in sport-induced tourism previously observed similar differences compared to their Western counterparts [21]. Participants’ comments included:

*“I did not belong in a predominantly white community. So that’s why I chose a community or a coworking space based on the community as I test it out. I tried out several coworking spaces, but I tested it out based on “how do I do as an Asian?”*

Another participant said:

*“Most of the places I was perhaps the only Indian who was traveling while working a lot of people told me that I was the only Indian, so having some friends from home along to accompany with me was not an option because I was the only one I knew who could do this.”*

The last theme is related to the importance of the first impression, so-called vibe, or energy of the place that comes mostly from people. Five participants recognized that they draw conclusions about the space from the first few moments in a place, and participants even compared it with the first day in school or mentioned that it is like love at first sight. This first impression was often studied in hospitality and was found to be valuable [22, 23]. However, in contrast with hotel experience, where the webpage is a contributor to the first impression image, all participants shared the importance of an actual appearance in the coworking and coliving to evaluate the people and atmosphere. This contribution highlights the influence of offline impression to understanding digital nomad experience and is aligned with the importance of co-creation and co-production in coworking spaces [5, 7].

## Conclusion

This paper contributed to the body of knowledge by identifying elements that are valued in coworking and coliving space during travel. The content analysis of semistructured interviews with digital nomads who stayed in coworking and coliving space assisted in disclosing their travel experience. As a result, a summary of the advantages and disadvantages of coworking and coliving space seen by digital nomads was developed (Table 1). This study discovered that digital nomads highly value community sense, pleasant work environment, convenience, ability to consult and learn from others; these factors are aligned with findings in the previous research [6, 16, 17, 19, 20]. Also, participants appreciated that coworking and coliving space helps them with local tips, like restaurants and things to do, especially during the first visit to the area. Disadvantages of coworking space indicated by participants include limited availability of coliving accommodation, loud parties, high prices compared to similar accommodation, and lack of privacy. Moreover, a new finding of the study is the acknowledgment of Asian nomads and their different perspectives of community experience with a mostly western accumulation of digital nomads.

Among the practical implications of this study, perhaps the most important one is that coworking and coliving space can be an attraction of destinations and serve as a peculiar tourism information center. It suggests to the tourism boards who focus on digital nomads to invest in relationships with coworking businesses, just as it is commonly practiced with theme parks and hotels. The findings may serve as a guideline for destinations and business owners to develop new products, adjust marketing strategy, and improve the service quality.

The limitation is related to the range of coliving spaces covered in the study. As community-based coliving may have different forms, there are unique retreat farms, converted Buddhist temples, community-based eco-villages, and many others. However, the study has initially reached out to travelers who stayed in establishments that call themselves a coliving space (as part of the coworking space) and focused the study around them. Future research is worth developing a categorization of coworking and coliving spaces for travelers and factors influencing a particular establishment’s choice during travels, such as length of stay and trip composition.

Out of the current study’s scope, all participants indicated that they were looking forward to their next adventure once travel restrictions are down; some of them even were still travelling during data collection (in May 2020) and stayed in the coworking and coliving space at the moment of the interview. Digital nomad tourism can continue to be the world’s significant phenomenon after the mobility change of COVID-19. More future research on understanding digital nomads as a segment for tourism is called for.
